# BMC Veterinary Research reviewer acknowledgement, 2013

**DOI:** 10.1186/1746-6148-10-28

**Published:** 2014-01-28

**Authors:** Hayley Henderson

**Affiliations:** 1 BioMed Central, Floor 6, 236 Gray’s Inn Road, London WC1X 8HB, United Kingdom

## Abstract

**Contributing reviewers:**

The editors of BMC Veterinary Research would like to thank all our reviewers who have contributed to the journal in Volume 9 (2012).

## 

Emmanuel Abatih

Belgium

Darrell Abernethy

South Africa

Getu Abraham

Germany

Zbigniew Adamiak

Poland

Berihun Afera

Ethiopia

Hiroyuki Akagi

Japan

Masood Akhtar

Pakistan

Munir Aktas

Turkey

Pablo Alarcon

UK

Sanja Aleksic-Kovacevic

Serbia

David Allred

USA

Adelaide Almeida

Portugal

Andre Almeida

Portugal

Covadonga Alonso

Spain

Silvia Alonso

UK

Flavio Ribeiro Alves

Brazil

Sharif Aly

USA

Monica Amarante

Brazil

Burim Ametaj

Canada

Hermann Ammer

Germany

Beatriz Amorena

Spain

Renee Amorim

Brazil

Arturo Anadon

Spain

Kevin Anderson

USA

Peter Angele

Germany

Rodrigo Angerami

Brazil

Muhammad Irfan Anwar

Pakistan

Toshiro Arai

Japan

Alicia Aranaz

Spain

Flábio Araújo

Brazil

David Argyle

UK

Yutaka Arimura

Japan

Fabrice Armougom

France

Julie Arsenault

Canada

Karin Artursson

Sweden

Lucy Asher

UK

Spiridoula Athanasiadou

UK

Robert Atterbury

UK

Ruben Avendaño-Herrera

Chile

Roger Ayling

UK

Shawn Babiuk

Canada

Alex Bach

Spain

Wolfgang Baeumer

USA

Maria Terezinha Bahia

Brazil

Árpád Csaba Bajcsy

Hungary

Jalal Bakhtiari

Iran

Vinayagamurthy Balamurugan

India

Alfonso Baldi

Italy

Anne Balkema-Buschmann

Germany

Hywel Ball

UK

Maria Ballester

Spain

Ashley Banyard

UK

Claudio Barbeito

Argentina

Anthony Barbet

USA

Zoe Barker

UK

Jason Bartz

USA

Anna Bassols

Spain

Max Bastian

Germany

John Bauer

USA

Claudia Baule

Sweden

Elisabeth Baum

USA

Maximilian Baumann

Germany

Ronald Baynes

USA

Jonathan Beever

USA

Andreas Beineke

Germany

Elsa Beltran

UK

David Bemis

USA

Dusan Bencina

Slovenia

Staffan Bensch

Sweden

Lise C. Berg

Denmark

Philip Bergman

USA

Olaf Berke

Canada

Dirk Berkvens

Belgium

Umberto Bernabucci

Italy

Giuseppe Bertoni

Italy

Giuseppe Bertoni

Switzerland

Daniela Betz

Germany

V Bhanuprakash

India

Rakesh Bhatnagar

India

Giacomo Biagi

Italy

Richard Bianco

USA

Erica Bickerton

UK

Dorothee Bienzle

Canada

Damer Blake

UK

Jose M Blasco

Spain

Gerd Bobe

USA

Patrick Boerlin

Canada

Peter Boettcher

Germany

Anette Boklund

Denmark

Yugendar Reddy Bommineni

USA

Patcharee Boonsiri

Thailand

Luke Borst

USA

Paolo Bosi

Italy

Adrian Boswood

UK

Barry Bradford

USA

Bruno Brasil

Brazil

Ana Bratanich

Argentina

Jackie Brearley

UK

Walter Brehm

Germany

Julia Breuer

Germany

Gert Breur

USA

Angela Briganti

Italy

Robert Briggs

USA

Susan Brockmeier

USA

Ellen Brooks-Pollock

UK

Scott Brown

USA

Glenn Browning

Australia

Jeffrey Bryan

USA

Bryce Buddle

New Zealand

Steven Budsberg

USA

Sava Buncic

Serbia

Dominik Burger

Switzerland

Eric Burrough

USA

Patrick Butaye

Belgium

Richard Butterwick

UK

Barbara Byrne

USA

Pedro Cabrales

USA

Luigi Calamari

Italy

Paolo Calistri

Italy

Henrik Callesen

Denmark

Tereza Cardoso

Brazil

James Carmalt

Australia

Librado Carrasco

Spain

Mícheál Casey

Ireland

Geovanni Cassali

Brazil

Rudi Cassini

Italy

Joaquin Castilla

Spain

Tatiana Castro

Brazil

Eleonor Castro-Janer

Uruguay

Adriano Casulli

Italy

Salvatore Catania

Italy

Lorenzo Cecconi

Italy

Fabrizio Ceciliani

Italy

Natalia Cernicchiaro

USA

Jose Ceron

Spain

Pablo Chacana

Argentina

Chanhee Chae Korea,

South

Mark Chambers

USA

Prem Chapagain

USA

Gilles Charpigny

France

Sylvie Chastant-Maillard

France

Kuldeep Chattha

USA

Elisabetta Chiaradia

Italy

Alan Chicoine

Canada

Munashe Chigerwe

USA

Michael Childress

USA

Jane Cho

USA

Ratan Choudhary

India

Tracy Clegg

Ireland

Craig Clifford

USA

Salvador Climent

Spain

Alison Clode

USA

Joan Coates

USA

Julio Coll

Spain

Nancy Cornick

USA

Marialaura Corrente

Italy

Pablo Corva

Argentina

Solenne Costard

UK

Mark Costello

New Zealand

Emmanuel Couacy-Hymann

Cote d'Ivoire

Gwen Covey-Crump

UK

Kevin Cummings

USA

Charles Czuprynski

USA

Aleksandro Da Silva

Brazil

Mauro Dacasto

Italy

Trachsel Dagmar

France

Janet Daly

UK

Escribano Damián

Spain

Josh Daniels

USA

Peter Davies

USA

Ujjwal De

India

Lucia De Almeida

USA

Steven De Decker

UK

Geoffrey De Lisle

New Zealand

Lieven De Zutter

Belgium

Chitrita Debroy

USA

Jeroen Degroot

Netherlands

Aldo Dekker

Netherlands

Frederic Delbac

France

Leonardo Della Salda

Italy

Matthew Denwood

UK

Nicolai Denzin

Germany

Nick Dervisis

USA

Amelie Desvars

France

Linda Detwiler

USA

Jeroen Dewulf

Belgium

Brian Diekman

USA

Linda Dixon

UK

Padraic Dixon

UK

Jane Dobson

UK

Klaus Doll

Germany

Lydia Donaldson

USA

Philippe Dorchies

France

Julian Drewe

UK

Bert Driessen

Belgium

Zhi-Qiang Du

USA

Edward Dubovi

USA

J Dudhia

UK

Birgitta Duim

Netherlands

William Dundon

Austria

Sophie Duraffour

Belgium

Sue Dyson

UK

David Eckersall

UK

Christina Eckstrand

USA

John Egan

Ireland

Agneta Egenvall

Sweden

Monika Egerbacher

Austria

Anna Vigdis Eggertsdottir

Nigeria

Brucw Eilts

Australia

Daniela Eisinger

Germany

Meike Eklund

Germany

Jennifer Elliman

Australia

Angela Ellis

USA

Wael El-Tras

Egypt

Suat Erdogan

Turkey

Gisela F Erf

USA

Nuria Eritja

Spain

Mark Erwin

Canada

Kevin Esch

USA

Sabine Essler

Austria

Christelle Fablet

France

Maria Fahie

USA

Virginia Fajt

USA

Joseph Falkinham

USA

Shufang Fan

USA

Timothy Fan

USA

Anna Maria Farca

Italy

Mark Farnworth

New Zealand

Wenche Farstad

Norway

Vahab Farzan

Canada

Folorunso Oludayo Fasina

South Africa

Francesco Fazio

Italy

Antonio Fernandez

Spain

Ezio Ferroglio

Italy

William Fielding

Bahamas

Veerle Fievez

Belgium

Natalie Finch

UK

Filomena Fiorito

Italy

Rebuma Firdessa

Germany

Simon Firestone

Australia

Zlata Flegar-Mestric

Croatia

Jason Folster

USA

Leila Fonseca

Brazil

Simone Forterre

Switzerland

Geoffrey Fosgate

South Africa

Caroline Fossum

Sweden

Derek Foster

USA

David Fox

USA

Leo Foyle

Australia

Lorenzo Fraile

Spain

Philippe Fravalo

Canada

Sara Frazier

USA

Linda Frellstedt

Belgium

Robert Friendship

Canada

Michael Fries

Germany

Jean-Pierre Frossard

UK

Sarah Gabriël

Belgium

Alba Galan

Spain

Marina Gallo Calderon

Argentina

Adelina Gama

Portugal

Stepan Gambaryan

Germany

Victor Gannon

Canada

Ana L. Garcia-Perez

Spain

Michael Gardner

USA

Laura Garrett

USA

Joseba M. Garrido

Spain

Raul Jaime Gazmuri

USA

Martina Gerken

Germany

Kerstin Gerlach

Germany

Wilhelm Gerner

Austria

William Gerthoffer

USA

Tilahun Getachew

Ethiopia

Joern Gethmann

Germany

Joachim Geyer

Germany

Abdullah Gharamah

Yemen

Mirutse Giday

Ethiopia

James Gilkerson

Australia

Pedro J. Ginel

Spain

Mario Giorgi

Italy

Jacques Godfroid

Norway

Ak Goel

India

Wilfred Goldmann

UK

Lorenzo Gonzalez

UK

Robert Goodband

USA

Jutta Gottschalk

Germany

Marcelo Gottschalk

Canada

Kevin Gough

UK

Sheryl Gow

Canada

Bruce Grahn

Canada

Jean-Guillaume Grand

France

Nicolas Granger

UK

Erik Granquist

Norway

Iain Grant

UK

Andrew Greer

New Zealand

Maria-Jesus Grilló

Spain

Felix Grimm

Switzerland

Mirjam Grobbel

Germany

Marvin J. Grubman

USA

Yi Guan

China

Sanjeev Gupta

Ireland

Silvina Gutiérrez

Argentina

Carlton Gyles

Canada

Thomas Hagenaars

Netherlands

Veronika Halas

Hungary

Ailsa Hall

UK

William Hall

Australia

David Hampson

Australia

Sanni Hansen

Denmark

Wendy Hartig-Merkel

Germany

Marlene Hauck

USA

Kevin Haussler

USA

Yamen Hegazy

Egypt

Nagendra Hegde

India

Marco Helder

Netherlands

Wouter Hendriks

Netherlands

Moon Her

Korea, South

Aureliano Hernandez

Colombia

Cord Heuer

New Zealand

Jorg Heukelbach

Brazil

Yoshiaki Hikasa

Japan

Mike Hildreth

USA

Gaby Hirsbrunner

Switzerland

Akihiko Hiyama

Japan

Gen Hiyama

Japan

Jean-François Hocquette

France

Douglas Hodgins

Canada

Margarethe Hoenig

USA

Ole Hojberg

Denmark

Dietmar Holm

South Africa

Lindsey Holmstrom

USA

Menno Holzhauer

Netherlands

Walther Honscha

Germany

Brian Hosie

UK

Margaret Hosie

UK

Mari Hovinen

Finland

Chienjin Huang

Taiwan

Korinna Huber

Germany

Eleonora Iacono

Italy

Yamamoto Ichiro

Japan

Yasuo Inoshima

Japan

Jo Ireland

UK

Hirotaka Iwase

Japan

Stine Jacobsen

Denmark

Magdalena Jacobson

Sweden

Mario Jacques

Canada

Fulton Janet

USA

Nick Jeffery

USA

Dean Jerry

Australia

Maria Vang Johansen

Denmark

Christopher Johnson

USA

Thora J. Jonasdottir

Norway

David Jordan

Australia

Tae Sung Jung

Korea, South

Christian Junghanss

Germany

Polona Juntes

Slovenia

Piotr Jurka

Poland

Witold Kędzierski

Poland

Hiroshi Kajigaya

Japan

Mehmet Kale

Turkey

Marissak Kalpravidh

Thailand

Bernhard Kaltenboeck

USA

Donata Kalthoff

Germany

Ji-Houn Kang

Korea, South

Narasimhan Kannan

Singapore

Jeffrey Kaplan

USA

Martin Kasny

Czech Republic

Philip Kass

USA

Sabine Kastner

Germany

Lisa Katz

Ireland

Frank Katzer

UK

Carol Kaufman

USA

Nigatu Kebede

Ethiopia

Stefan Keller

USA

Michael Kent

USA

Arifa Khan

USA

Camilla Kielland

Norway

Manfred Kietzmann

Germany

Amy Kilbride

UK

Nuh Kilic

Turkey

Wolfgang Klee

Germany

Don Klinkenberg

Netherlands

Robert Klopfleisch

Germany

Nobuo Koizumi

Japan

Svend Kold

UK

Ganesh Kondabattula

India

Dean Konjevic

Croatia

Timm Konold

UK

Polychronis Kostoulas

Greece

Krzysztof Kostro

Poland

Magdalena Krol

Poland

Oliver Krone

Germany

Kate Kukanich

USA

Janina Kulka

Hungary

Rajesh Kumar Vaid

India

Tetsuo Kunieda

Japan

Keiichi Kuroki

USA

Ohkyeong Kweon

Korea, South

Trine L'Abee-Lund

Norway

Marcelo Labruna

Brazil

Elizabeth Laird

UK

Luke Simon Lambeth

Australia

Susan Lana

USA

Jan Langeveld

Netherlands

Sylvain Larrat

Canada

Jennifer Larsen

USA

Lubomir Lashev

Bulgaria

Susanna Lau

Hong Kong

Richard Laven

New Zealand

Vladimir Lazarevic

Switzerland

Marie-Frederique Le Potier

France

Peter Leegwater

Netherlands

Sigrid Lehnert

Australia

Andrea Lengeling

UK

Erin Leonard

Canada

Veronique Leray

France

Marie Lewis

UK

Yvan L'Homme

Canada

Bin Li

China

Mingwei Li

China

Guodong Liang

China

Ai-Ho Liao

Taiwan

Walter Lilenbaum

Brazil

Min Lin

Canada

Annette Litster

USA

Jianzhu Liu

China

Qinfang Liu

USA

Catherine Logue

USA

David Logue

UK

Cheryl London

USA

Paul Loomis

USA

Ermias Lulekal

Ethiopia

Oliver Lung

Canada

Amandine Lurette

France

Thomas Lutz

Switzerland

Konstantin Lyashchenko

USA

Inna Lysnyansky

Israel

Felipe Maciel

Brazil

Janet Macinnes

Canada

Ben Maddison

UK

Sally Madsen-Bouterse

USA

Akihiko Maeda

Japan

Rafael Maertínez-Díaz

Spain

Dominiek Maes

Belgium

Erin Malone

USA

Panagiotis Mantis

UK

Denis Marcellin-Little

USA

Silvana Marchioro

Brazil

Lieta Marinelli

Italy

Stanley Marks

USA

Inmaculada Martin-Burriel

Spain

Jorge Martinez

Spain

Beatriz Martinez-Lopez

Spain

Beatriz Martínez-López

Spain

Silvia Martinez-Subiela

Spain

Daniele Martins

Brazil

Christiane Massicotte

USA

Candace Mathiason

USA

Gift Matope

Zimbabwe

Augusto Matos

Portugal

Sandro Mazzariol

Italy

Kathleen Mc Entee

Belgium

Tim Mcallister

Canada

Dominic Mccafferty

UK

Sean Mcdonough

USA

Colin Mcinnes

UK

Gerry Mclachlan

UK

Mary-Louise Mclaws

Australia

Don Mcmanus

Australia

James Mcnair

UK

Julie Meachen

USA

Graham Medley

UK

Els Meeusen

Australia

Matthew Mellema

USA

Paula Menzies

Canada

Kristen Messenger

USA

Asla Mete

USA

Raul Miguez-Lozano

Spain

Djordje Miljkovic

Serbia

Woutrina Miller

USA

Rowan Milner

USA

J. Ernest Minton

USA

Carla Miranda

Portugal

Keiko Miyadera

USA

Shusei Mizushima

Japan

Mariko Mochizuki

Japan

Jaime Modiano

USA

Lars Moe

Norway

Jeffrey Mogil

Canada

Matlou Mokgotho

South Africa

Jan Mol

Netherlands

Susana Monteiro

Portugal

Gabriel Moran

Chile

Simon More

Ireland

Marcella Mori

Belgium

Nobuko Mori

Japan

Erich Möstl

Austria

Rinaldo Mota

Brazil

Luca Motta

UK

Martha Mulks

USA

Thomas Muller

Germany

Tamás Müller

Hungary

Peter Mullowney

Ireland

John Muma

Zambia

Karen Munana

USA

Hetron Munang'Andu

Norway

Yoshihiro Muneta

Japan

Lina Mur

Spain

Shelton Murinda

USA

Pamela Murison

UK

David Murphy

UK

Sue Murphy

UK

Jane Murray

UK

Jo Murrell

UK

Michael Murtaugh

USA

Hanspeter Naegeli

Switzerland

Hiromichi Nagasawa

Japan

Miho Nagasawa

Japan

Vinny Naidoo

South Africa

Munekazu Naito

Japan

Kristina Narfstrom

USA

Lubna Nasir

UK

Clive Naylor

UK

Haileleul Negussie

Belgium

Eric Nelson

USA

Laura Nelson

USA

Mandy Nevel

UK

Richard Newton

UK

Ana Maria Nicola

Argentina

Andrew Niehaus

USA

Jens Peter Nielsen

Denmark

Soeren Saxmose Nielsen

Denmark

Nathan Nieto

USA

Theo Niewold

Belgium

Noriyuki Nishida

Japan

Koji Nishifuji

Japan

Chiara Noli

Italy

Tim Nuttall

UK

Robert O'Brien

USA

Matjaz Ocepek

Slovenia

Annette O'Connor

USA

Rosangela Odore

Italy

Javier Oliveira Alvarez

Spain

Teresa Cristina Oliveira-Sequeira

Brazil

Sigvard Olofsson

Sweden

Pal A. Olsvik

Norway

Dan O'Neill

UK

Maarten Oosterlinck

Belgium

Tanja Opriessnig

UK

Geert Opsomer

Belgium

Toomas Orro

Estonia

Angel Ortiz-Pelaez

UK

Christopher Orton

USA

Leigh Owens

Australia

Mark Oyama

USA

Tarja Pääkkönen

Finland

Justin Pachebat

UK

Akos Pakozdy

Austria

Saverio Paltrinieri

Italy

Sameer Dinkar Pant

Denmark

Sophia Papageorgiou

USA

Mark Papich

USA

Roberto Amerigo

Papini Italy

Bart Pardon

Belgium

Bongkyun Park

Korea, South

Se Chang Park

Korea, South

Elizabeth Parker

Australia

Clare Partridge

UK

Frank Pasmans

Belgium

David Pearl

Canada

Niels Pedersen

USA

Luc Peelman

Belgium

Ming Pei

USA

Ludovic Pelligand

UK

Greg Penner

Canada

Dominique Penninck

USA

Louis Penning

Netherlands

Felipe Perecin

Brazil

Andrew Peregrine

Canada

José Pérez

Spain

Rob Pettitt

UK

Wolfram Petzl

Germany

Ute Philipp

Germany

Christine Piek

Netherlands

Sofie Piepers

Belgium

Alda Pires

USA

Patrick Pithua

USA

Gerry Polton

UK

Iuliana Popa

France

Thibaud Porphyre

UK

Simon Pot

Switzerland

Keith Poulsen

USA

Antonio Pozzi

USA

John Prescott

Canada

Peter Presek

Germany

Anna Puigdemont

Spain

Margo Pybus

Canada

Satu Pyörälä

Finland

Valeria Quattrocchi

Argentina

Ahmad Rabiee

Azerbaijan

Heidi Radke

UK

Emily Radlowski

USA

De Maria

Raffaella Italy

Md Rahman

USA

Swaraj Rajkhowa

India

Alejandro Ramirez

USA

Ana S. Ramirez

Spain

Carlos Alberto Ramos

Brazil

Peter Ramzan

UK

Neeraj Rana

India

Geertrui Rasschaert

Belgium

Ralf Regenthal

Germany

Scott Reid

UK

Christopher Reilly

USA

Luis Carlos Reis

Brazil

Xiaofeng Ren

China

David Rendle

UK

David Renter

USA

Gourapura Renukaradhya

USA

Jesus Requena

Spain

Jack Rhyan

USA

Kristy Richards

USA

Kimberly Riehle

USA

Gerald Rimbach

Germany

Espen Rimstad

Norway

Robert Ringseis

Germany

Rebecca Robinson

UK

Carlos Rodriguez

USA

Alexander Rodriguez-Palacios

USA

Bernard Roelen

Netherlands

Vanessa Rohlf

Australia

Jesus L Romalde

Spain

Mariarita Romanucci

Italy

Paola Roncada

Italy

Vibeke Rootwelt

Norway

Edoardo Rosato

Italy

Luca Rossi

Italy

Sophie Rossi

France

Tanya Rossi

Canada

Alessandra Rota

Italy

Joseph Rubin

Canada

Stephen Russell

USA

Thomas Rutkowski

USA

Daniel Ruzek

Czech Republic

Andrew Rycroft

UK

Marie-Pierre Ryser-Degiorgis

Switzerland

Simo Saarakkala

Finland

Konrad Sachse

Germany

Marc Saez

Spain

Irene Salinas

USA

Siba Samal

USA

Ahmed Samir

Egypt

Javier Sanchez

Canada

Juan Sanhueza

New Zealand

Andreia Santos

Portugal

Berta São Braz

Portugal

Jan M Sargeant

Canada

Giuseppe Sarli

Italy

Rosa Elena Sarmiento-Silva

Mexico

Tomohiro Sasanami

Japan

Mamoru Satoh

Japan

Jimmy Saunders

Belgium

Carola Sauter-Louis

Germany

Jason Sawyer

UK

Eugenio Scanziani

Italy

Rüdiger Schade

Germany

David Schaeffer

USA

Esther Schelling

Switzerland

Martin Schmidt

Germany

Jonas Schmidt-Chanasit

Germany

Sandra Scholes

UK

Gerald Fritz Schusser

Germany

Heinzpeter Schwermer

Switzerland

Phil Scott

UK

Fernanda Seixas Travassos

Portugal

Ian Self

UK

Hiroshi Sentsui

Japan

Branka Seol Martinec

Croatia

Hans-Martin Seyfert

Germany

Tizhong Shan

China

Tongling Shan

China

Patrick Shearer

USA

Quan Shen

China

Andre Shih

USA

Ronald Shikiya

USA

Kinji Shirota

Japan

Victoria Siarkou

Greece

Nagini Siddavaram

India

Chummy Sikalizyo Sikasunge

Zambia

Hubert Simhofer

Austria

Ellen Singer

UK

Silvia Siso

USA

Janusz Skierski

Poland

Katherine Skorupski

USA

Brigita Slavec

Slovenia

Graham Smith

UK

Hilde Smith

Netherlands

Richard Smith

UK

Stephanie Smith

USA

Laura Soler Vasco

Belgium

Ulrike Sorge

USA

Smaragda Sotiraki

Greece

Esteban Soto

Saint Kitts and Nevis

Andrew Sparkes

UK

Stephen Spatz

USA

Subramaniam Srikumaran

USA

Francesco Staffieri

Italy

Karl Stahl

Sweden

Katharina Stärk

UK

Carsten Staszyk

Germany

Christoph Staubach

Germany

Jenny Stavisky

UK

Frank Steffen

Switzerland

Barry Stein

USA

Timothy Stein

USA

Joshua Stern

USA

Kim Stevens

UK

Matthew Stewart

USA

Andrej Steyer

Slovenia

Mark Stidworthy

UK

Karl Stoffel

Switzerland

Jeffrey Stott

USA

George Strain

USA

Viola Strompfová

Slovakia

Christina Strube

Germany

Henrik Stryhn

Canada

Subbaya Subramanian

USA

Jan Suchodolski

USA

Shazia Sultana

Malaysia

Jane Sykes

USA

Wing-Kin Syn

UK

Bozena Szafranska

Poland

Christoph Szober

Germany

Fern Tablin

USA

Ryo Tadano

Japan

Hiromi Takahashi-Omoe

Japan

Rachel Tan

Australia

Xun Tan

China

Saraya Tavornpanich

Norway

Polly Taylor

UK

Francisco Teixeira-Neto

Brazil

Fabrice Teletchea

France

Erik Teske

Netherlands

Caroline Tessier

France

Jens Tetens

Germany

Eileen Thacker

USA

Larissa Thackray

USA

Douglas Thamm

USA

Mohamed Tharwat

Egypt

Betty Theriault

USA

Francois Thiaucourt

France

Etienne Thiry

Belgium

John Thomason

USA

Peter Thompson

South Africa

Kevin Thorneloe

USA

Laurence Tiley

UK

Tijs Tobias

Netherlands

Christopher Todd

UK

Herbert Tomaso

Germany

Laura Tomassone

Italy

Guang-Zhi Tong

China

Montserrat Torremorell

USA

Samia Toukhsati

Australia

Pierre-Louis Toutain

France

Imke Traulsen

Germany

Henry Tremaine

UK

Erminio Trevisi

Italy

Eric Troncy

Canada

Gregory Troy

USA

Marianna Tryfonidou

Netherlands

Rea Tschopp

Switzerland

Panagiotis Tsikouras

Greece

Changchun Tu

China

Julia Tünsmeyer

Germany

Asta Tvarijonaviciute

Spain

Hermann Unger

Austria

Mark Ungrin

Canada

Melissa Upjohn

UK

Åse Uttenthal

Denmark

Francisco Uzal

USA

Wilfried Vahjen

Germany

Emanuela Valle

Italy

Eric Van Breda

Netherlands

Henri Van Bree

Belgium

Gerlinde Van De Walle

USA

Emmanuelle Van Erck

Belgium

Ed Van Klink

UK

Ariette Van Knegsel

Netherlands

Katrien Vanderperren

Belgium

Alain Vanderplasschen

Belgium

An Vanhaesebrouck

UK

Estelle Venter

South Africa

Kata Orsolya Veres-Nyéki

Hungary

Helene Verheije

Netherlands

Kristien Verheyen

UK

Subhash Verma

India

David Verner-Jeffreys

UK

Haralabos Ververidis

Greece

Lene Karine Vestby

Norway

Aldo Vezzoni

Italy

Joaquin Vicente

Spain

Martin Vidal

USA

Carlos

Portugal

Natassia Vieira

USA

Jose Manuel Vilar

Spain

Maria J Vilar

Finland

Cecilia Villaverde

Spain

Willem Vink

New Zealand

Charles Vite

USA

Lieven Vlaminck

Belgium

Holger Volk

UK

Wilna Vosloo

Australia

Katleen Vranckx

Belgium

Andy Wales

UK

Per Wallgren

Sweden

Gemma Walmsley

UK

Donal Walsh

USA

Raquel Walton

USA

Ching-Ho Wang

Taiwan

Hongning Wang

China

Wycliffe Wanzala

Kenya

Cerian Webb

UK

Cynthia Webster

USA

Scott Weese

Canada

Glen Weiser

USA

Alexander Weiss

Germany

William Weiss

USA

Hsin-Yi Weng

USA

Philip Wertz

USA

Caroline Wheeler-Jones

UK

Christopher Whipps

USA

Phillip Whitfield

UK

Ted Whittem

Australia

Paul Wichgers Schreur

Netherlands

Barbara Wieland

Mongolia

Marcy Wilder

Japan

Scott Willard

USA

Kim Willoughby

UK

Stephen Wilson

Belgium

Wayne Wilson

Australia

Klaus Wimmers

Germany

Jack Windig

Netherlands

Helen Winefield

Australia

Anna Winnicka

Poland

Min-Liang Wong

Taiwan

Michal Wróbel

Poland

Panagiotis Xenoulis

USA

Ebru Yalcin

Turkey

Chih-Wei Yang

Taiwan

Hanchun Yang

China

Kui Yang

USA

Rong Ye

China

Carl Yeoman

USA

Han Sang Yoo

Korea, South

Yasunaga Yoshikawa

Japan

Jesse Young

USA

Bjørnar Ytrehus

Norway

Li Yu

China

Kai Zacharowski

Germany

Valentina Zappulli

Italy

Daniel Zarski

Poland

Jürgen Zentek

Germany

Zhidong Zhang

Canada

Feng-Qi Zhao

USA

Jeff Zimmerman

USA

Jakob Zinsstag

Switzerland

Annetta Zintl

Ireland

Feng-Cai Zou

China

Martín José Zumárraga

Argentina

